# Montelukast reduces seizures in pentylenetetrazol-kindled mice

**DOI:** 10.1590/1414-431X20155031

**Published:** 2016-02-23

**Authors:** J. Fleck, F.R. Temp, J.R. Marafiga, A.C. Jesse, L.H. Milanesi, L.M. Rambo, C.F. Mello

**Affiliations:** 1 Departamento de Fisiologia e Farmacologia, Centro de Ciências da Saúde, Universidade Federal de Santa Maria, Santa Maria, RS, Brasil; 2 Hospital Universitário de Santa Maria, Universidade Federal de Santa Maria, Santa Maria, RS, Brasil; 3 Departamento de Farmácia, Centro de Ciências da Saúde, Centro Universitário Franciscano, Santa Maria, RS, Brasil

**Keywords:** Montelukast, CysLT1R, CysLT2R, Seizure, PTZ, Kindling

## Abstract

Cysteinyl leukotrienes (CysLTs) have been implicated in seizures and kindling; however, the effect of CysLT receptor antagonists on seizure frequency in kindled animals and changes in CysLT receptor expression after pentylenetetrazol (PTZ)-induced kindling have not been investigated. In this study, we evaluated whether the CysLT1 inverse agonist montelukast, and a classical anticonvulsant, phenobarbital, were able to reduce seizures in PTZ-kindled mice and alter CysLT receptor expression. Montelukast (10 mg/kg, *sc*) and phenobarbital (20 mg/kg, *sc*) increased the latency to generalized seizures in kindled mice. Montelukast increased CysLT_1_ immunoreactivity only in non-kindled, PTZ-challenged mice. Interestingly, PTZ challenge decreased CysLT_2_ immunoreactivity only in kindled mice. CysLT_1_ antagonists appear to emerge as a promising adjunctive treatment for refractory seizures. Nevertheless, additional studies are necessary to evaluate the clinical implications of this research.

## Introduction

Epilepsy is a chronic neurological disease characterized by recurrent seizures due to excessive discharge of cerebral neurons, and by emotional and cognitive dysfunction (1). This disorder affects approximately 50 million individuals worldwide and at least 30% of patients remain refractory, despite the use of antiepileptic drugs (2). Considering the high proportion of patients who do not respond to available treatment, it is essential to search for novel therapeutic targets and to identify seizure mechanisms.

Several lines of evidence indicate that inflammation plays a role in epilepsy. Experimental and clinical studies have shown that seizures induce brain inflammation and recurrent seizures perpetuate chronic inflammation (3,4). Indeed, arachidonic acid (AA) is released from membrane phospholipids during seizures, and oxidized by COX (cyclooxygenase) and LOX (lipoxygenase), generating AA proinflammatory products (5). The products of this "uncontrolled arachidonic acid cascade" include prostaglandins, thromboxanes and leukotrienes. Levels of prostaglandin, and leukotriene B_4_ and C_4_ are increased in the hippocampus of epileptic patients and in the cerebrospinal fluid of children with febrile seizures (6,7). In addition, kainic acid-induced seizures are associated with increased brain levels of leukotrienes and PGF_2α_ in the cortex, hippocampus and hypothalamus of rats (8). In accordance with these findings, a role for leukotriene receptors, particularly of the CysLT_1_ subtype, has been proposed in seizure/epilepsy (8 9 10 11). Although LTD_4_ (a CysLT_1_ receptor agonist) facilitates pentylenetetrazol (PTZ)-induced seizures, intracerebroventricular (*icv*) injection of montelukast (a CysLT_1_ receptor inverse agonist) decreases PTZ-induced seizures. In addition, *icv* montelukast prevents PTZ-induced blood-brain barrier (BBB) disruption and leukocyte infiltration (10), and potentiates the anticonvulsant effect of phenobarbital on PTZ seizures and decreases sedation, a major side effect of phenobarbital (11). Montelukast attenuates PTZ-induced myoclonic jerks and increases oxidative stress markers in rats (12). However, it is still unknown whether CysLT_1_ receptor antagonism reduces seizures in animals with established seizure susceptibility, such as kindled animals. Therefore, the aim of the current investigation was to evaluate whether montelukast (a CysLT_1_ inverse agonist) reduces seizures in PTZ-kindled mice. The effects of pharmacological treatment, kindling, and challenge with PTZ on CysLT_1_ and CysLT_2_ receptor immunoreactivity in the cerebral cortex of mice were also examined.

## Material and Methods

### Animals

Young male Swiss mice (25-28 g, 42 days old) from the Animal House of the Universidade Federal de Santa Maria, Santa Maria, RS, Brazil, were used. Animals were housed 12 in an acrylic cage (35 × 52 × 17 cm) under controlled light and environmental conditions (12/12 h light/dark cycle, 22±1°C, 55% relative humidity). Food (Supra, Brazil) and drinking water were provided *ad libitum*. Behavioral tests were carried out during the light cycle from 9:00 to 17:00 h, in accordance with the national and international legislation (Guidelines of the Brazilian Council of Animal Experimentation - CONCEA - and the EU Directive 2010/63/EU for animal experiments). The protocols were designed to minimize the number of animals used, as well as their suffering, and were approved by the Committee on Care and Use of Experimental Animal Resources of the Universidade Federal de Santa Maria (authorization No. 084/2013).

### Reagents

PTZ was purchased from Sigma-Aldrich (USA), LTD_4_ and montelukast were from Cayman Chemical (USA), and phenobarbital was from Cristália Pharmaceutical Co. (Brazil). PTZ was dissolved in isotonic saline (0.9% NaCl). Phenobarbital and montelukast were dissolved in 0.5% dimethyl sulfoxide and sterile apyrogenic saline containing 10% propylene glycol. Fresh drug solutions were prepared immediately before use.

### Kindling induction and seizure observation

Mice were intraperitoneally (*ip*) injected with saline (10 ml/kg) or PTZ (35 mg/kg) three times a week (Monday, Wednesday, and Friday) for 5 weeks, followed by an application-free interval of 1 week (13). After each PTZ injection, convulsive behavior was observed for 20 min and classified into the following stages, as described by Ferraro et al. (14): stage 0, no behavioral change; stage 1, hypoactivity and immobility; stage 2, two or more isolated, myoclonic jerks; stage 3, generalized clonic convulsions with preservation of righting reflex; and stage 4, generalized clonic or tonic-clonic convulsions with loss of righting reflex.

**Figure 1:**
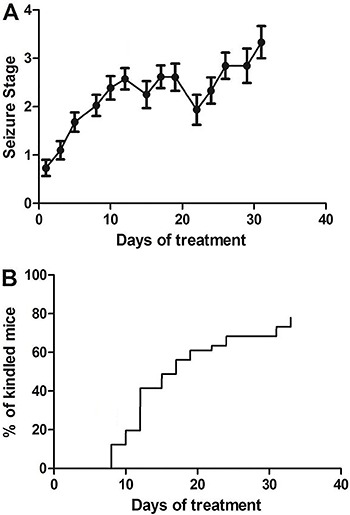
Time-course for induction of kindling (n=32) after repeated pentylenetetrazol (35 mg/kg, *ip*) administration. Results are reported as medians and interquartile range of seizure stage (*A*) and the cumulative percentage of the total number of kindled mice (*B*).

An animal was considered kindled when it displayed stage 3 or 4 seizures in three consecutive sessions. The mean time to kindling was 11.2±1.3 days. Overall, 70% of the mice were kindled, 20% were not, and 7% died. Figure 1A and B shows the time-course for effective induction of kindling.

**Figure 2:**
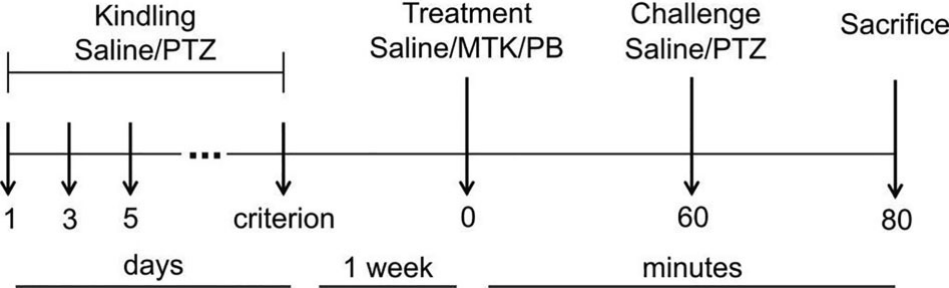
Experimental protocol. Animals were injected with pentylenetetrazol (PTZ) (35 mg/kg, *ip*) on days 1, 3, 5, 8, 10, 12, 15, 17, 19, 22, 24, 26, 29, 31, and 33 until a specific criterion was met. Each kindled mouse was matched with a saline-treated animal. One week after kindling induction, mice were treated with saline, MTK (10 mg/kg, *ip*) or PB (20 mg/kg, *ip*), 60 min before challenge with PTZ (35 mg/kg, *ip*) or saline (*ip*). Animals were observed for 20 min and sacrificed. PTZ: pentylenetetrazol; MTK: montelukast; PB: phenobarbital.

The animals that reached kindling criterion were kept drug-free for 1 week and injected subcutaneously (*sc*) with montelukast (10 mg/kg, *sc*), phenobarbital (20 mg/kg, *sc*), or saline (10 mg/kg, *sc*). After 60 min, the animals were challenged with PTZ (35 mg/kg, *ip*) or saline. Mice were monitored by video for 20 min, and the latency to myoclonic jerks and generalized tonic-clonic seizures were recorded. Mice were sacrificed by decapitation at the end of the observation period. The cerebral cortex was quickly removed and stored at -80°C until processing. As expected, animals challenged with saline did not exhibit seizures. Therefore, these animals were not included in the behavioral analysis. For each kindled animal, a saline-treated animal with the same number of injections was assigned to the same pharmacological treatment, and subjected to challenge with saline or PTZ. Figure 2 shows the full experimental design, with the 12 resulting groups, and Table 1 displays the frequency of seizures in the challenge session.

**Table:**
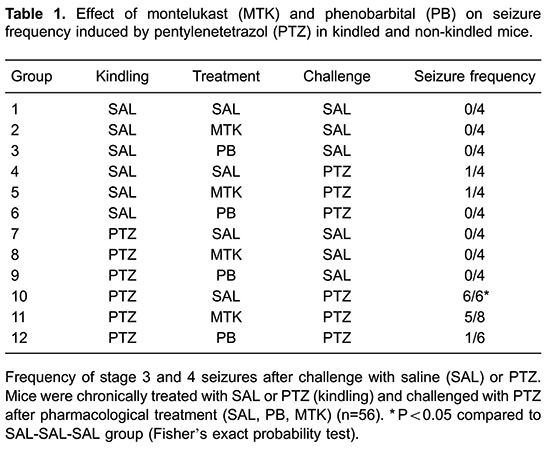

### Western blot

All Western blot procedures were conducted as described by Guerra et al. (15). The cerebral cortex was homogenized in a cold (4°C) lysis buffer containing 10 mM HEPES, pH 7.9, 10 mM KCl, 2 mM MgCl_2_, 1 mM EDTA, 1 mM NaF, 1 mM phenylmethanesulfonyl fluoride, 10 mM β-glycerophosphate, 1 mM DTT and 2 mM sodium orthovanadate, and a mixture of protease and phosphatase inhibitors (Sigma-Aldrich). The homogenates were centrifuged (12,700 *g*) for 30 min at 4°C and the supernatant (S1), denominated cytosolic fraction, was reserved for posterior processing. The pellet (P1) was resuspended in lysis buffer with 1% Triton-X, incubated for 15 min in ice, and centrifuged at 12,700 *g* for 60 min at 4°C. The supernatant (S2), containing the membrane fraction, was collected for subsequent analysis and the pellet (P2) was stored at -80°C. The protein concentration in the membrane fraction was measured with the bicinchoninic acid assay using bovine serum albumin (BSA) as a standard. The supernatant proteins (20 μg) were resolved by polyacrylamide gel electrophoresis (SDS-PAGE) and electroblotted onto nitrocellulose membranes (Millipore, USA). Membranes were blocked with 5% BSA in TBS-T (0.05% Tween 20 in Tris-borate saline) plus 5% non-fat milk at room temperature for 1 h, then incubated overnight at 4°C with primary antibodies: rabbit anti-CysLT_1_R (1:5000, Santa Cruz Biotechnology, USA) or goat anti-CysLT_2_R (1:5000, Santa Cruz Biotechnology). This procedure was followed by incubation with horseradish peroxidase-conjugated secondary antibodies (1:3000, Santa Cruz Biotechnology) at room temperature for 3 h. Blots were developed by enhanced chemiluminescence (ECL; Thermo Fisher Scientific, USA) and the band intensities were quantified by ImageJ 219 (NIH). In these experiments, β-actin (1:50000, Santa Cruz Biotechnology) was used as an internal reference. The results were normalized for densitometry values in the control group (saline-saline-saline) and reported as the relative amount of CysLT_1_R, CysLT_2_R. Proteins were probed in the same membranes after stripping with 0.5 M NaCl in 0.2% SDS/TBS at 60°C for 50 min.

### Statistical analysis

Latency to myoclonic jerks and generalized tonic-clonic seizures were analyzed by two-way ANOVA for nonparametric data (Ray-Scheirer-Hare test followed by Mann-Whitney test, with Bonferroni's correction for multiple comparisons). These data are presented as the medians and interquartile range. Western blots were analyzed by a factorial 2 (saline or PTZ - "kindling") × 3 (saline, montelukast or phenobarbital - "treatment") × 2 (saline or PTZ - "challenge") ANOVA, followed by Bonferroni's test, and are reported as means ± SEM. P<0.05 was considered to be significant.

## Results

### Seizure evaluation

**Figure 3:**
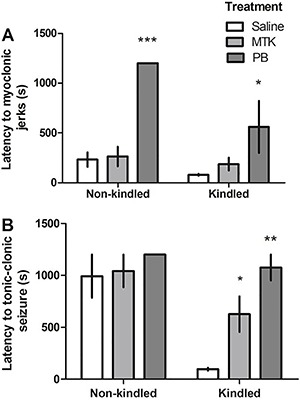
Effect of montelukast (MTK) and phenobarbital (PB) on pentylenetetrazol-induced seizures (35 mg/kg, *ip*). *A*, Latency to myoclonic jerk; B, latency to tonic-clonic generalized seizure. Data are reported as medians and interquartile ranges for n=4-8 per group. A probability of P<0.05 was considered to be significant. *P<0.05, **P<0.01 and ***P<0.001, compared to the saline group (Mann-Whitney test, with Bonferroni's correction).

Figure 3 shows the effects of montelukast (10 mg/kg, *sc*) and phenobarbital (20 mg/kg, *sc*) on PTZ-induced seizures (35 mg/kg, *ip*), measured as the latency to myoclonic jerk (A) and latency to generalized tonic-clonic seizures (B). Phenobarbital increased the latency to myoclonic jerks in kindled and non-kindled animals [H(2)=19.3; P<0.01]. Both phenobarbital and montelukast increased the latency to generalized seizures in kindled animals [H(2)=19.0; P<0.01].

### Western blot analysis

**Figure 4:**
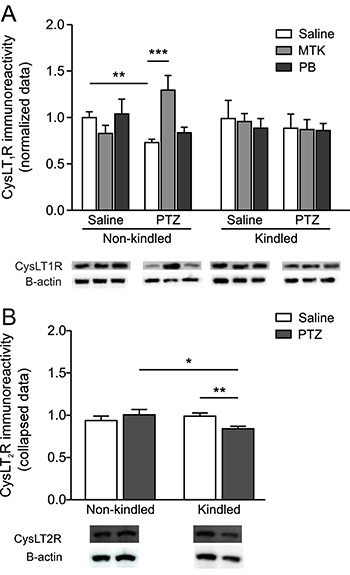
Effect of pentylenetetrazol (PTZ) kindling on CysLT_1_R (*A*) and CysLT_2_R (*B*) immunoreactivity in the cortex. Mice were treated with saline, montelukast (MTK) or phenobarbital (PB) and challenged or not with PTZ. Representative blots are shown below each group. Representative immunoblots shown in panel B are from saline-injected animals. Data are reported as means ± SEM for n=3-5 per group, from 5 different experiments. *P<0.05, **P<0.01, ***P<0.001 (Bonferroni's *post hoc* test).

Figures 4A and B show the effects of kindling, pharmacological treatment (saline, montelukast or phenobarbital), and challenge with PTZ (or saline) on CysLT_1_ and CysLT_2_ receptor immunoreactivity in the cerebral cortex, respectively. Statistical analysis revealed a significant kindling (saline or PTZ) by treatment (saline, montelukast or phenobarbital) by challenge (saline or PTZ) interaction [F(2,38)=3.71; P=0.034; η^2^=0.16] for CysLT_1_ (Figure 4A). *Post hoc* analysis revealed that while PTZ challenge reduced CysLT_1_R immunoreactivity in non-kindled animals that received saline, it increased CysLT_1_R immunoreactivity in non-kindled mice that received montelukast. Pharmacological treatment and PTZ challenge did not alter CysLT_1_ receptor immunoreactivity in the cortex of PTZ-kindled mice.

Statistical analysis of CysLT_2_ receptor immunoreactivity revealed a significant kindling (saline or PTZ) by challenge (saline or PTZ) interaction [F(1,38)=5.81; P=0.021; η^2^=0.13] (Figure 4B). *Post hoc* analysis revealed that montelukast decreased CysLT_2_ immunoreactivity only in non-kindled animals that were not challenged with PTZ. In other words, kindling and PTZ challenge abolished montelukast-induced decreases in CysLT_2_ receptor immunoreactivity.

## Discussion

In this study, montelukast and phenobarbital reduced seizure frequency in PTZ-kindled mice. Montelukast administration increased CysLT_1_ immunoreactivity only in non-kindled PTZ-challenged mice. Interestingly, PTZ challenge decreased CysLT_2_ immunoreactivity only in kindled mice.

These findings are in agreement with the current view that CysLT_1_ inverse agonists decrease seizures (10,11), and extend from previous data showing that systemic montelukast impairs kindling induction with PTZ (9). It has recently been demonstrated that the CysLT_1_ inverse agonist montelukast synergistically increases the anticonvulsant action of phenobarbital against PTZ-induced seizures. Moreover, LTD_4_, a cysteinyl leukotriene, reverses the effect of montelukast (11). Indeed, epilepsy is associated with increased levels of inflammatory mediators in the brain, including leukotrienes, which are produced by neurons, glia, and endothelial cells in the BBB (16,17). BBB dysfunction may result from brain insults such as status epilepticus or traumatic brain injury (18), and evidence suggests that it may facilitate epileptogenesis or even aggravate the epileptic condition (19). Increased BBB permeability can persist for several weeks, months or even years, and this may contribute to enhanced excitability, possibly due to brain inflammation (20). In line with this view, single (21) and repeated administration of chemoconvulsant agents, such as PTZ, enhance BBB permeability (22). The brain areas most affected by PTZ-induced BBB disruption are the hypothalamus and cerebellum (21). Neutrophils that have breached the BBB can lead to the immediate synthesis of cysteinyl leukotrienes (CysLTs). These pro-inflammatory mediators derived from the AA 5-lipoxygenase pathway (23) are involved in various diseases, including asthma, cerebral ischemia and brain trauma (24 25 26). CysLTs significantly increase after fluid percussion-induced brain injury, being detected as early as 10 min after injury and continuing to rise over an hour (27,28).

Despite convincing evidence suggesting that CysLT_1_R antagonism maintains BBB integrity (29), which is a possible mechanism of seizure protection, pharmacological data provided by Lenz et al. (10) indicate that additional mechanisms may underlie the anticonvulsant effect of montelukast. In accordance, Palmer et al. (30) demonstrated that LTD_4_ increases the firing rate of Purkinje cells *in vivo*, suggesting an excitatory role for this lipid mediator.

Two aspects of the present study are particularly significant from the translational point of view. The first is that systemic administration of montelukast reduced seizure frequency in kindled mice. The second is that montelukast is currently used in the clinic to treat asthma (31). Therefore, concerns about the toxicity of montelukast in humans or the need for unusual administration routes (usually *icv* in preclinical studies) that could limit its clinical use do not apply (10,11). Previous studies have shown that acute systemic administration of montelukast does not decrease seizures in mice (9). This is similar to unpublished data from our group and other studies indicating that systemic montelukast does not prevent PTZ-induced seizures in mice, as well as evidence that montelukast and pranlukast cross the BBB poorly. Therefore, it appears that the anticonvulsant effect of montelukast depends on previous BBB disruption, which occurs in both kindling and epilepsy. This is in full agreement with a study reporting that pranlukast increases the anticonvulsant efficacy of a number of classic anticonvulsants in patients with intractable partial epilepsy (32).

In this study, we also showed that while PTZ challenge decreased, montelukast increased CysLT_1_R immunoreactivity in non-kindled mice. These findings are, to some extent, similar to the findings of Dupré et al. (33) who demonstrated that while montelukast, MK571 and zafirlukast (inverse agonists of CysLT_1_R) increase, LTD_4_ decreases cell surface receptor expression in COS-7 cells. Agonist binding to a G-protein coupled receptor enables receptor phosphorylation and interaction with beta-arrestin, leading to receptor sequestration from the cell surface (34), making it available to proteolytic cleavage. Accordingly, inverse agonists may stabilize the active receptor on the cell surface and interfere with the internalization process (33). Although the membrane surface content of CysLT1 receptors was not assessed in this study, it is reasonable to assume that LTD_4_ decreased total CysLT1 immunoreactivity by facilitating receptor internalization and proteolysis. In line with this view, Li *et al*. (35) have shown that only inverse agonists were able to block internalization and down-regulation of opioid receptors. In addition, as expected, montelukast did not alter CysLT_2_ receptor immunoreactivity, indicating selectivity of the inverse agonist towards CysLT_1_ receptors. It is important to emphasize that neither the anticonvulsant effect of montelukast nor the anticonvulsant effect of phenobarbital depended on alterations in CysLT_1_ immunoreactivity, because CysLT_1_ immunoreactivity was not altered in kindled animals.

In contrast to the CysLT_1_ receptor, PTZ-induced challenge decreased CysLT_2_ receptor immunoreactivity only in kindled animals. These results suggest that kindling may have distinct effects on the response of CysLT_1_ and CysLT_2_ receptors to PTZ challenge. Because montelukast-induced effects on CysLT_1_ immunoreactivity were also impaired in kindled animals, it may be proposed that kindling impairs CysLT_1_, but facilitates CysLT_2_ adaptive responses. Interestingly, chemical kindling increases NR2A subunit mRNA in the hippocampus, γ2 subunit of GABA_A_ receptor mRNA in the piriform cortex (36), and GABA_B_ receptor binding of whole brain (37). These neurochemical alterations may reflect the neuronal loss and synaptic reorganization that occurs in PTZ-kindled animals, and are accompanied by an increase in the immunoreactivity of glial fibrillary acid protein, a marker of astrocytes (38), suggesting reactive gliosis. In addition to neuronal cell loss and gliosis, PTZ kindling induces mossy fiber sprouting (39) and sprouting in the CA1 and the subiculum of rats (40). However, kindling itself did not alter CysLT receptor immunoreactivity in our experimental conditions. Given the multiplicity of cellular alterations observed in this model, further experiments, designed to study the expression patterns and internalization dynamics of CysLT receptors in different cell types after kindling, should be performed to clarify the effects of kindling on CysLT receptors and to determine if they play a role in seizure facilitation.
